# Ultrastructural Evidence of Synapse Preservation and Axonal Regeneration Following Spinal Root Repair with Fibrin Biopolymer and Therapy with Dimethyl Fumarate

**DOI:** 10.3390/polym15153171

**Published:** 2023-07-26

**Authors:** Paula Regina Gelinski Kempe, Mateus Vidigal de Castro, Victor Campos Khuriyeh, Benedito Barraviera, Rui Seabra Ferreira, Alexandre Leite Rodrigues de Oliveira

**Affiliations:** 1 Laboratory of Nerve Regeneration, Department of Structural and Functional Biology, Institute of Biology, University of Campinas, Campinas 13083-862, SP, Brazil; paulakempe@gmail.com (P.R.G.K.); mateusvidigal@hotmail.com (M.V.d.C.); v225097@dac.unicamp.br (V.C.K.); 2 Center for the Study of Venoms and Venomous Animals (CEVAP), São Paulo State University (UNESP), Botucatu 18610-307, SP, Brazil; benedito.barraviera@unesp.br (B.B.); rui.seabra@unesp.br (R.S.F.J.)

**Keywords:** spinal cord, motoneuron, avulsion, fibrin biopolymer, synapse

## Abstract

Spinal cord injury causes critical loss in motor and sensory function. Ventral root avulsion is an experimental model in which there is the tearing of the ventral (motor) roots from the surface of the spinal cord, resulting in several morphological changes, including motoneuron degeneration and local spinal cord circuitry rearrangements. Therefore, our goal was to test the combination of surgical repair of lesioned roots with a fibrin biopolymer and the pharmacological treatment with dimethyl fumarate, an immunomodulatory drug. Thus, adult female Lewis rats were subjected to unilateral ventral root avulsion of L4–L6 roots followed by repair with fibrin biopolymer and daily treatment with dimethyl fumarate (15 mg/Kg; gavage) for 4 weeks, the survival time post-surgery being 12 weeks; n = 5/group/technique. Treatments were evaluated by immunofluorescence and transmission electron microscopy, morphometry of the sciatic nerve, and motor function recovery. Our results indicate that the combination between fibrin biopolymer and dimethyl fumarate is neuroprotective since most of the synapses apposed to alfa motoneurons were preserved in clusters. Also, nerve sprouting occurred, and the restoration of the ‘g’ ratio and large axon diameter was achieved with the combined treatment. Such parameters were combined with up to 50% of gait recovery, observed by the walking track test. Altogether, our results indicate that combining root restoration with fibrin biopolymer and dimethyl fumarate administration can enhance motoneuron survival and regeneration after proximal lesions.

## 1. Introduction

Brachial and lumbar plexuses injuries occur in high-energy trauma, such as car accidents, falls from heights, and extreme sports. Due to the great amplitude of movement in the shoulder joint, the cervical and upper thoracic roots are more susceptible to traction forces and rupture. In turn, spinal motoneurons present in the ventral horn are axotomized significantly close to the cell body, resulting in retrograde degeneration, gliosis, and inflammation of the gray matter. Attempts to restore the avulsed roots started about fifty years ago [1], and, although some positive outcomes have been obtained by Carlsted and colleagues [2,3], the investigation of the cellular and molecular aspects of the injury remain key elements for the development of more effective therapeutic approaches. For that, the establishment of animal models of root avulsion allowed the advance in understanding the several reactions to proximal axotomy as well as aspects of the regeneration process [4,5,6,7,8,9,10,11]. Thus, ventral root avulsion (VRA) in rats has become an animal model widely used since it can recapitulate both acute and chronic phases following injury [3,12,13,14,15,16,17,18,19,20,21].

In the acute phase following VRA, motoneurons display stereotyped morphological changes due to disconnection between the cell body and its target. Such interruption of axonal continuity results in metabolic and electrophysiological changes in the cell soma, well-known as chromatolysis [22]. Together with that, robust modifications in the spinal circuitry take place. A hallmark is the retraction of pre-synaptic inputs to alpha motoneurons, with a preferential loss in excitatory glutamatergic terminals, which originate mostly from the dorsal root ganglia conveying proprioceptive information [11,23,24,25]. Nevertheless, the remaining inhibitory boutons from interneurons [26,27] seem to contribute to a firing resting state of the axotomized motoneurons so that the metabolism is focused on cell repair and regrowth.

In the long term, such changes determine whether the injured neurons survive or degenerate, which has been interpreted as a window of opportunity for the application of neuroprotective therapies. In turn, the use of neurotrophic molecules, such as GDNF and BDNF, can increase the survival rate of avulsed motoneurons [28]. However, antioxidant and anti-inflammatory drugs show advantages regarding systemic administration over longer periods without strong side effects. In this line of research, we have shown the positive effects of dimethyl fumarate daily administration over four weeks after ventral root avulsion and reimplantation. Neuroprotection, downregulation of glial reaction, and motor recovery were obtained up to 12 weeks survival time, reinforcing the concept that acute pharmacological treatment is crucial for long-term recovery [29]. Herein, we show ultrastructural evidence that DMF therapy preserves inputs to alpha motoneurons, which in turn correlates with the regrowth and myelination of large axons of the sciatic nerve, and motor recovery over time. As a result, functional recovery was achieved, indicating a possible translation to the clinic.

## 2. Materials and Methods

### 2.1. Experimental Animals and Groups

This study was approved by the Institutional Committee for Ethics in Animal Experimentation (Committee for Ethics in Animal Use—Institute of Biology—CEUA/IB/UNICAMP, proc. Nr. 4500-1, Campinas, SP, Brazil). All experiments were performed by the guidelines of the Brazilian College for Animal Experimentation. Forty adult 8-week-old female Lewis rats (LEW/HsdUnib) were housed with free access to food and water in a 12 h light/dark cycle. The animals were subjected to unilateral ventral root avulsion of the lumbar L4, L5, and L6 roots. Twelve weeks after injury, the animals were euthanized; the lumbar spinal cords and sciatic nerves were processed for light and transmission electron microscopy.

The animals were divided into 4 groups: (1) ventral root avulsion without reimplantation and vehicle treatment (VHL); (2) ventral root avulsion without reimplantation and treatment with dimethyl fumarate—15 mg/kg (DMF); (3) ventral root avulsion followed by root repair with fibrin biopolymer and vehicle treatment (FB+VHL); and (4) ventral root avulsion followed by root repair with fibrin biopolymer and treatment with DMF—15 mg/kg (FB+DMF; n = 10 for all groups). The contralateral (CTL) sciatic nerve and spinal cord side were used as the control. 

DMF (242926-100 g, Sigma-Aldrich, Waltham, MA, USA) was diluted in 0.08% methylcellulose solution and administered daily for 4 weeks after injury at a dose of 15 mg/kg by gavage [29]; methylcellulose solution was administered as the vehicle. Both treatments started 30 min after the surgical procedures. Fibrin biopolymer derived from the venom of Crotalus durissus terrificus (Cdt), combined with buffalo fibrinogen, was applied at the injury site as follows: 3 μL of fibrinogen-rich cryoprecipitate derived from buffalo blood, 2 μL of calcium chloride, and 1 μL of a thrombin-like enzyme-enriched fraction extracted from Cdt venom [30,31,32,33,34,35]. FB components and application formulas are listed in its patent (BR1020140114327) and were kindly provided by the Center for the Study of Venoms and Venomous Animals (CEVAP, Brazil).

### 2.2. VRA and Lesioned Motor Root Reimplantation

Animals were induced to anesthesia with a combination of xylazine hydrochloride (Anasedan^®^, 10 mg/kg, Sespo Indústria e Comércio, Paulínia, SP, Brazil) and ketamine hydrochloride (Dopalen^®^, 50 mg/kg, Sespo Indústria e Comércio, Paulínia, SP, Brazil) at a ratio of 1:1.5, applied intraperitoneally. After loss in reflexes, the animals were kept in deep anesthesia with isoflurane (2%). A lubricating ophthalmic gel (Liposic, 2 mg/g; 0.2% *w/w*) was applied to the eyes, and the animals were kept on a heated pad during the surgical procedure.

To assess the spinal cord, the rat was put in a prone position, the back was shaved, and an incision was made parallel to the vertebral column from the lower thoracic region to the upper lumbar segment. The musculature was released for visualization of the thoracic and lumbar vertebrae. A laminectomy of approximately two vertebrae was performed (T13-L1) so that the lumbar intumescence was exposed. The dissection of the dura mater and the denticulate ligament was performed. After exposing the spinal cord, the ventral roots associated with lumbar segments were exposed. Avulsion was performed unilaterally on the ventral lumbar roots referring to L4, L5, and L6 spinal segments with the aid of fine forceps. The animals in the reimplanted groups had the ventral lumbar roots repositioned to their original sites before avulsion immediately after the injury and reimplanted using FB. The stability of the root fixation was observed to evaluate the success of the repair. After the surgical procedures, the spinal cord roots were repositioned to their original position, and the musculature, fascia, and skin were sutured in layers.

Surgical recovery was performed in a postsurgical environment with heating for 4 h. Tramadol hydrochloride (Germed Farmacêutica Ltda, Hortolândia, SP, Brazil) was administered at a dose of 0.5 mg/kg by gavage every 24 h until the third postoperative day. If any signs of pain (prostration, ruffled fur, decreased food consumption) and discomfort were detected, the administration of tramadol hydrochloride was extended appropriately.

### 2.3. Specimen Preparation

Twelve weeks post-lesion, animals were euthanized with a lethal dose of a combination of xylazine hydrochloride (Anasedan^®^, 30 mg/kg, Sespo Indústria e Comércio, Paulínia, SP, Brazil) and ketamine hydrochloride (Dopalen^®^, 150 mg/kg, Sespo Indústria e Comércio, Paulínia, SP, Brazil) at a ratio of 1:1.5 applied intraperitoneally, and the vascular system was transcardially perfused.

For immunofluorescence analysis, after rinsing with buffered saline (PB), animals were perfused with 4% formaldehyde in PB 0.1 M (pH 7.4). The lumbar intumescence and a 1 cm mid-thigh segment of the sciatic nerve (ipsi and contralateral) were collected and postfixed for 24 h at 4 °C in the same fixative. They were then kept in a solution of 10%, 20%, and 30% sucrose for 24 h each at 4 °C. Samples were embedded in Tissue-Tek (Miles Inc., Oro Valley, AZ, USA) and frozen at −35 to −40 °C. Twelve micrometer-thick cross-sections of the spinal cord and longitudinal sections of the sciatic nerves were obtained in a cryostat (Micron HM25) and subsequently stored at −20 °C until use.

For morphometry and transmission electron microscopy, phosphate buffer 0.1 M (pH 7.4) saline was perfused through the left ventricle, followed by Karnovski fixative solution (glutaraldehyde 2% + paraformaldehyde 1%) in the same buffer (pH 7.4). The lumbar spinal cord and a 1 cm mid-thigh segment of the sciatic nerve (ipsi and contralateral) were collected and stored for 7 days in the same fixative solution at 4 °C. The specimens were then trimmed into smaller segments and separated between contra and ipsilateral, osmicated (3 h for nerve segments and 4 h for spinal cord segments), dehydrated through an ethanol series, and embedded in Durcupan ACM (Fluka, Steinheim, Switzerland).

### 2.4. Immunofluorescence

The slides containing the spinal cord and sciatic nerve cross-sections were initially acclimatized, washed with phosphate buffer (0.01 M CP) for 3 × 5 min, and incubated with blocking solution (bovine serum albumin—3% BSA + PB 0.1 M) for 45 min. Then, the slides were incubated for 3 h with anti-synaptophysin, anti-neurofilament, and anti-NeuN antibodies (Table 1) in an incubation solution (BSA 1% + Triton 0.2% + PB 0.1 M) and kept in a humid chamber at room temperature. They were then washed with PB 0.01 M (3 × 5 min), and the respective secondary antibodies were added (Alexa 488 or 594—1:400) and diluted in incubation solution for 45 min. Slides were washed once more in PB 0.01 M (3 × 5 min) and mounted in glycerol + DAPI (1:1000)/PB 0.01 M (3:1).

Immunostained specimens were observed and photographed under a fluorescence microscope (Leica DM 5500B, Wetzlar, Germany) coupled to a high-sensitivity camera (Leica DFC-345FX) at 20× or 40× magnification, depending on the analysis; three representative images were obtained for each animal on each side (injured/ipsilateral and uninjured/contralateral) of lamina IX of Rexed at the spinal cord ventral column and the sciatic nerve.

For quantification, the integrated density of pixels was measured, which represents the intensity of immunostaining, using ImageJ software (version 1.51 h). For all images at 20× magnification, the entire image was used for analysis, which is indicative of the microenvironment; for the anti-synaptophysin marker, the analysis was performed around the Neu-N-positive neurons at 40× magnification to assess the synaptic preservation of each motoneuron. The results are expressed as the ratio of the ipsilateral/contralateral side for each animal. 

### 2.5. Sciatic Nerve Regeneration

Resin blocks containing sciatic nerve segments were trimmed, and semithin sections (500 nm) were obtained in an ultramicrotome (Leica Ultracut UCT Ultramicrotome) and stained with Sudan black for examination under light microscopy (Leica DM 5500B). Each nerve was photographed at 10× magnification to obtain the total area using ImageJ software (version 1.51 h). Then, we obtained 6 images per animal at 100× magnification, sampling at least 15% of each nerve cross-section. These images were used for counting and measuring myelin fibers (Adobe Photoshop CS4 extended, Adobe Systems Incorporated, San Jose, CA, USA). For that, the following parameters were used: external diameter—myelin fiber diameter (FD), internal diameter—axon diameter (AD), myelin sheath thickness (MST), and ‘g’ ratio (GR), which is the ratio of axon diameter divided by the fiber diameter and indicates the degree of myelination (normal-, hypo-, or hyper-myelination).

### 2.6. Synaptic Analysis of the Ultrathin Sections

The resin blocks containing spinal cord segments were trimmed, and semithin sections (500 nm) were obtained in an ultramicrotome (Leica Ultracut UCT Ultramicrotome) and stained with toluidine blue for examination under light microscopy (Leica DM 5500B) to identify the presence of motor neurons at lamina IX of Rexed. Then, ultrathin sections (70 nm) from the L4–L6 segments were collected on formvar-coated single-slot copper grids, contrasted with uranyl acetate 4% and lead citrate, and examined under a Tecnai G2 Spirit BioTwin (FEI, Eindhoven, The Netherlands) transmission electron microscope operating at 80 KV. This procedure was performed for the ipsilateral side of all groups; the contralateral side used as the control was obtained from the VHL group.

Neurons with large cell bodies in the nuclear plane were identified as α-motoneurons by the presence of at least one C-type (cholinergic) nerve terminal. The neuronal membrane of these cells was then sequentially digitized at a magnification of 11,000× using a video camera (Eagle, FEI, Eindhoven) connected to a computerized system. The images were then mounted together in Corel Draw software (version 19), and the total perimeter of the neurons was measured using ImageJ software (version 1.51 h). 

The synaptic terminals apposing the motoneuron membrane were identified and classified as inhibitory (F-type—with flattened synaptic vesicles), excitatory (S-type—with spherical synaptic vesicles), and cholinergic (C-type), identified by a subsynaptic cistern with continuity with the rough endoplasmic reticulum [36,37]. After classification, they were counted and measured using the ImageJ program (version 1.51 h) to quantify synaptic coverage and proportion of buttons/100 μm of membrane; the distance between consecutive nerve buttons was also measured (GAPs). In total, two neurons per animal in the CTL, VHL, and DMF groups (n = 10 neurons for each group) and four neurons per animal in the FB+VHL and FB+DMF groups (n = 20 neurons for each group) were examined.

### 2.7. Motor Function Recovery Assessment

To evaluate the recovery of motor function, the Catwalk System (CatWalk XT 5.0, Noldus, Leesburg, VA, USA) was used [29,38]. The CatWalk system allows the detailed evaluation of gait recovery by using illuminated footprints and provides data such as size, pressure, width, and length of paw prints. One baseline motor measurement (before injury) was obtained as normal values for each animal. After the injury, assessments were performed every 18 days for 12 weeks. In each evaluation, 4 sessions were recorded per animal. An average was calculated for each baseline value and each of the days analyzed per animal and then for each experimental group ± standard error of the mean (SEM).

### 2.8. Statistical Analysis

From the numerical results from all the analyses, the mean and standard deviation were calculated for each group, and Prism 6 software was used. Immunofluorescence, nerve area, and myelin fiber count data were evaluated by the analysis of variance method—one-way ANOVA followed by Tukey’s post-test. Myelinated fiber morphometry data were analyzed using frequency distribution. The functional analysis data (walking track test) were evaluated by the analysis of variance method—two-way ANOVA and Mann–Whitney. Significance levels were assumed when * *p* < 0.05, ** *p* < 0.01, *** *p* < 0.001, and **** *p* < 0.0001 for all analyses.

## 3. Results

### 3.1. Synapse Preservation after Ventral Root Avulsion and DMF Treatment

To evaluate the overall synaptic preservation 12 weeks after avulsion/repair of the motor nerve roots, an anti-synaptophysin antibody was used. Photomicrographs were taken in the region of Rexed’s lamina IX at 20× magnification to analyze the microenvironment and 40× magnification to analyze the surface of the motoneurons. Immunostaining revealed baseline reactivity for the contralateral side of all groups for both magnifications.

**Figure 1:**
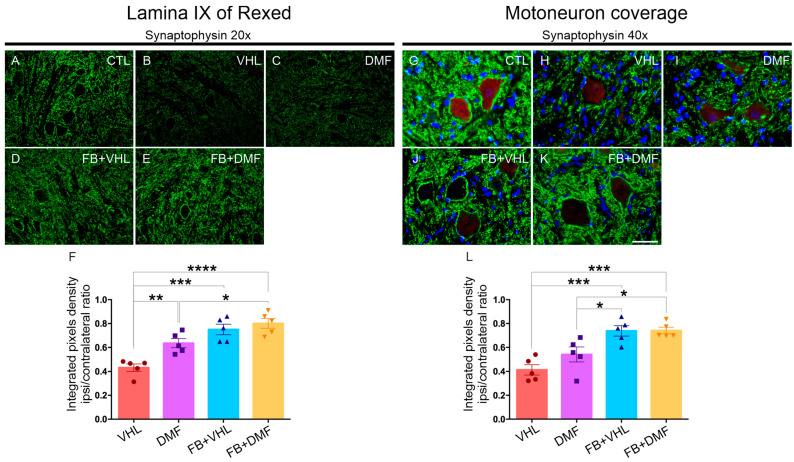
Immunostaining analysis of the neuropile and motoneuron coverage with anti-synaptophysin at lamina IX of Rexed 12 weeks postinjury. (**A**–**E**) Neuropile microenvironment analysis of all groups; groups that received reimplantation or DMF showed significant preservation of labeling intensity when compared to the group treated with vehicle alone. (**F**) Quantification (ipsi/contralateral ratio) of labeling, 12 weeks after ventral root avulsion. 20× magnification; scale bar = 100 μm. (**G**–**K**) Motoneuron coverage analysis in all groups showing root reimplantation is effective in preserving the spinal circuitry in apposition to motoneurons. (**L**) Synaptophysin motoneuron coverage quantification (ipsi/contralateral ratio), 12 weeks after motor root avulsion. 40× magnification; scale bar = 50 μm. N = 5. Mean values per group ± standard error of the mean. * *p* < 0.05, ** *p* < 0.01, *** *p* < 0.001 and **** *p* < 0.0001.

We observed lower synaptic density (40% decrease) after injury, indicating an intense retraction of terminals in apposition to axotomized motoneurons both in the neuropile and on the cell surface. DMF treatment provided synaptic preservation in the neuropile (63%) but not at the motoneuron surface level (54%) since coverage analysis showed no difference with the VHL group. However, reimplantation of lesioned roots significantly improved preservation (70%) both in the microenvironment and on the motoneuron surface (Figure 1, Table 2).

### 3.2. Ultrastructural Analysis of Alpha Motoneurons

**Figure 2:**
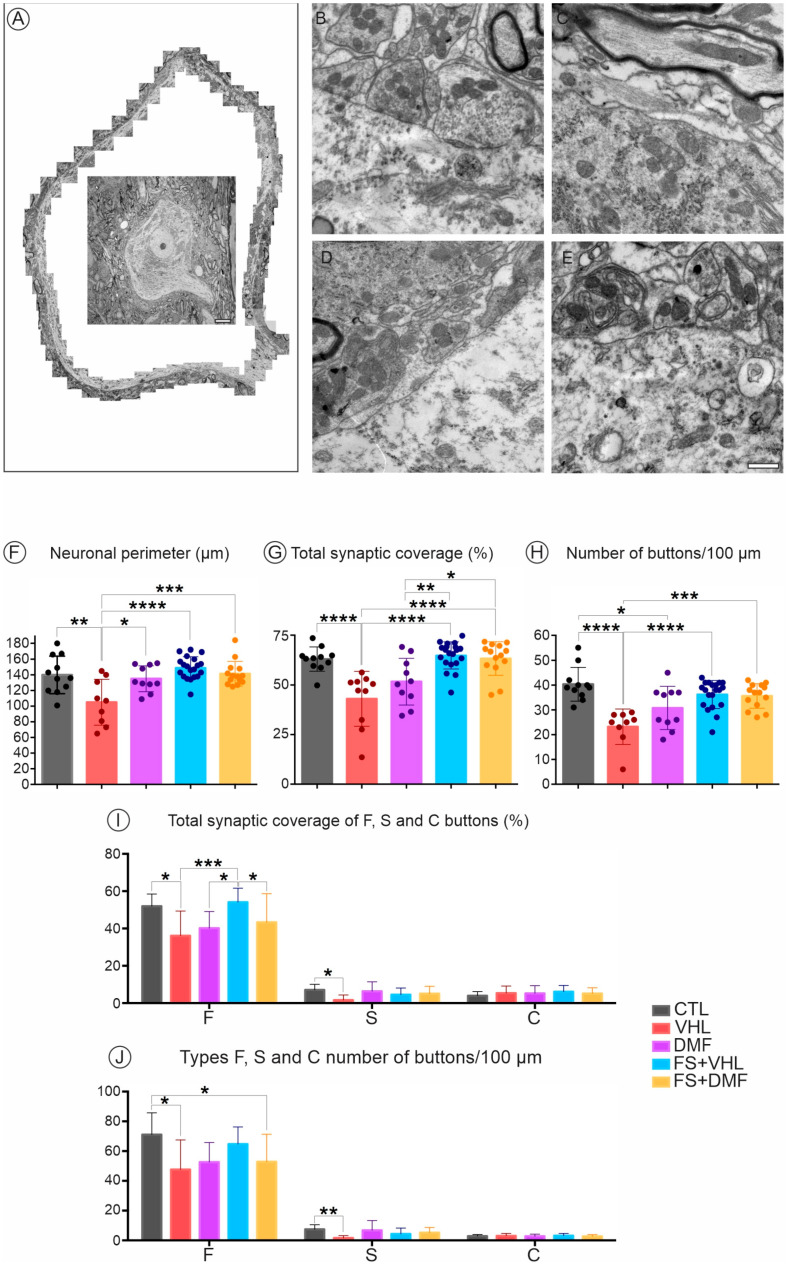
Analysis of the alpha motoneuron coverage, 12 weeks after injury. (**A**) Ultrastructural reconstruction of the membrane surface of an alpha-motoneuron present in the lumbar intumescence. Scale = 5 μm. (**B**) Normal pre-synaptic bouton apposition to the motoneuron membrane surface. (**C**) Synaptic loss following ventral root avulsion. (**D**,**E**) Representative examples of partially retracted boutons following motoneuron axotomy. (**F**) Neuronal perimeter. (**G**) Total synaptic coverage. (**H**) Number of buttons/100 μm of neuronal membrane. (**I**) Total synaptic coverage of Type F, S, and C buttons. (**J**) Types F, S, and C number of buttons/100 μm of neuronal membrane. N = 5. Mean values per group ± standard error of the mean. Scale bar = 500 nm. * *p* < 0.05, ** *p* < 0.01, *** *p* < 0.001 and **** *p* < 0.0001.

Ultrastructural analysis of the alpha motoneurons, present in the CTL group, revealed the expected perimeter of the neuronal membrane (140 μm on average), combined robust synaptic coverage (63%), and a significant proportion of synaptic buttons per 100 μm of membrane (approximately 40 buttons in apposition to the soma membrane). In the groups without repair of the lesioned root, we observed changes in the ultrastructure of the neuronal coverage of alpha motoneurons, where the presynaptic terminals were partially or completely retracted. We observed many glial projections, identified as astrocytes due to their low electron density, also in contact with the neuronal membrane, which filled the space between presynaptic terminals and the postsynaptic membrane. After injury, there was a reduction in neuronal perimeter in the VHL group (105 μm). For all groups that received any of the interventions, we observed that the perimeter was close to the value observed in the contralateral group, being 135 μm for the DMF group, 148 μm for the FB+VHL group, and 141 μm for the FB+DMF group. For synaptic coverage, there was an intense loss after axotomy, only 43% remaining in the VHL group and 51% in the DMF group when compared to the CTL group (63%). However, both groups that had their roots reimplanted, FB+VHL and FB+DMF, showed preservation of synaptic coverage, which was greater than 60%. A similar result was observed about the number of buttons apposed to every 100 μm of the neuronal membrane. After injury, the VHL group displayed an average of 23 buttons, and the DMF group showed about 30 buttons. Both groups submitted to root reimplantation preserved more than 35 buttons, which was closer to the values of the CTL group (40 buttons on average) (Figure 2, Table 3).

Furthermore, we obtained the synaptic coverage values and the number of buttons/100 μm for each type of terminal (F, S, and C). After injury, we observed a reduction in the synaptic coverage of F-type inhibitory terminals in all groups analyzed, except in the FB+VHL group. Interestingly, this group was similar to the CTL group. Regarding the S-type excitatory terminals (glutamatergic), we observed a reduction in synaptic coverage only in the VHL group when compared to all other groups. C-type cholinergic terminals showed no differences among groups. For the number of normalized buttons per 100 μm of neuronal membrane, we observed a reduction in synaptic coverage of F-type terminals after injury in the VHL and the FB+DMF groups. Both groups that received the therapies separately showed no differences from the CTL group. For S-type terminals, we observed a reduction only in the VHL group when compared to all other groups. Again, C-type cholinergic terminals showed no differences among groups (Figure 2, Table 4).

### 3.3. Nerve Terminals Distribution

**Figure 3:**
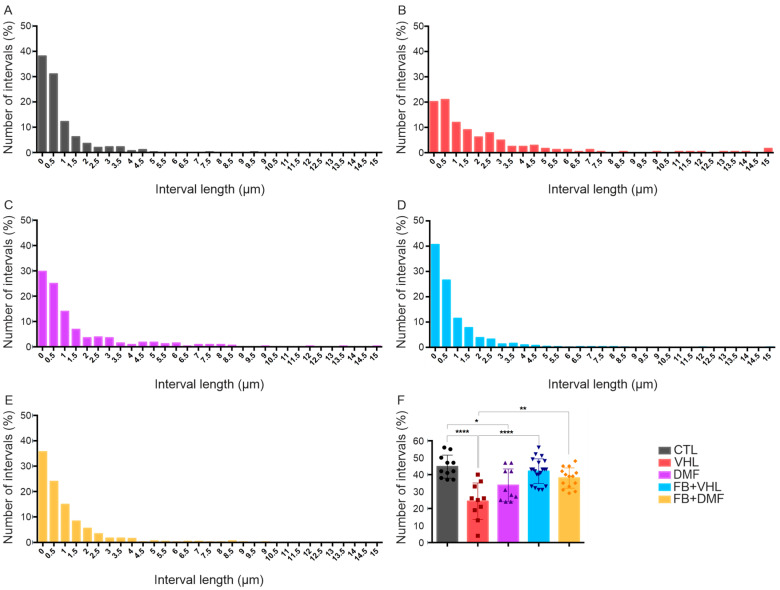
Consecutive space distribution (GAPs) between synaptic terminals along the neuronal membrane. Distribution values vary by 0.5 μm. (**A**) Normal distribution of intervals between nerve terminals—CTL group. (**B**–**E**) Distribution of intervals between nerve terminals in all experimental groups. (**F**) Number of intervals (GAPs) for each group. Mean values per group ± standard error of the mean. * *p* < 0.05, ** *p* < 0.01 and **** *p* < 0.0001.

The gaps between the terminals were measured, and their frequency distribution in 0.5 μm intervals was evaluated. These intervals were identified as filled with astroglial projections due to the low electron density. In normal neurons (CTL group), the terminals were grouped, with a large number of smaller intervals between the inputs, the largest being 4.5 μm. However, in the VHL group, a reduction in smaller intervals and an increase in the frequency of longer gaps between terminals were observed, with the longest gaps exceeding 15.0 μm. The DMF group presented intervals that increased to 8.5 μm. The FB groups, with lesioned root reimplanted, presented intervals very close to the CTL group (Figure 3).

After the injury (VHL group), a reduction in the number of GAPs was observed, with approximately 24 GAPs, and, for the DMF group, approximately 33 GAPs. However, we observed a greater number of GAPs in the CTL group (42 GAPs) and in the groups that were reimplanted, with 42 GAPs for the FB+VHL group and 38 GAPs for the FB+DMF group. These values indicate a better distribution of clusters of synaptic buttons after root reimplantation and DMF therapy (Figure 3).

### 3.4. Neurons with Incomplete Regeneration

**Figure 4:**
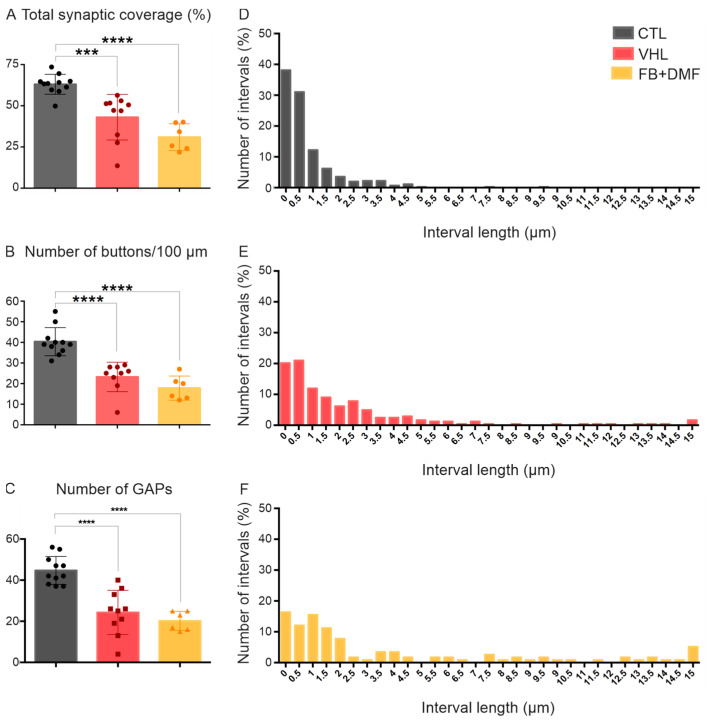
Incomplete or insufficient motoneuron regeneration. Analysis of surface ultrastructure and synapses of alpha-motoneurons on lamina IX of Rexed, 12 weeks after injury, regarding (**A**) total synaptic coverage, (**B**) number of buttons every 100 μm of neuronal membrane, (**C**) total number of intervals and (**D**–**F**) distribution of spaces (GAPs) between consecutive synaptic terminals along the neuronal membrane. Distribution values vary by 0.5 μm. Mean values per group ± standard error of the mean in (**A**–**C**). *** *p* < 0.001 and **** *p* < 0.0001.

Throughout the present study, we observed that many motoneurons in the FB+DMF group, which received both therapies, showed very similar characteristics to those of the VHL group. Thus, the regeneration of these neurons was incomplete or insufficient, possibly blocked by the glial scar. Synaptic coverage in motoneurons in the CTL group was 63%, while, in the VHL group, it was 43%. For neurons that showed insufficient regeneration, synaptic coverage was about 30%. Furthermore, the normalization of synaptic buttons per 100 μm of the neuronal membrane was also greatly reduced. For the CTL group, we observed an average of 40 buttons; for the VHL group, we observed 23 buttons; and, for neurons with insufficient regeneration, we observed only 17 buttons per 100 μm of the neuronal membrane. These results align with the distribution and size of gaps (GAPs) between synaptic terminals, where we observed that these neurons presented a distribution similar to that of the VHL group and were significantly different from the CTL group. There was a great decrease in the frequency of small intervals and an increase in the number of larger intervals, with several exceeding 15.0 μm (Figure 4, Table 5).

### 3.5. Nerve Preservation

**Figure 5:**
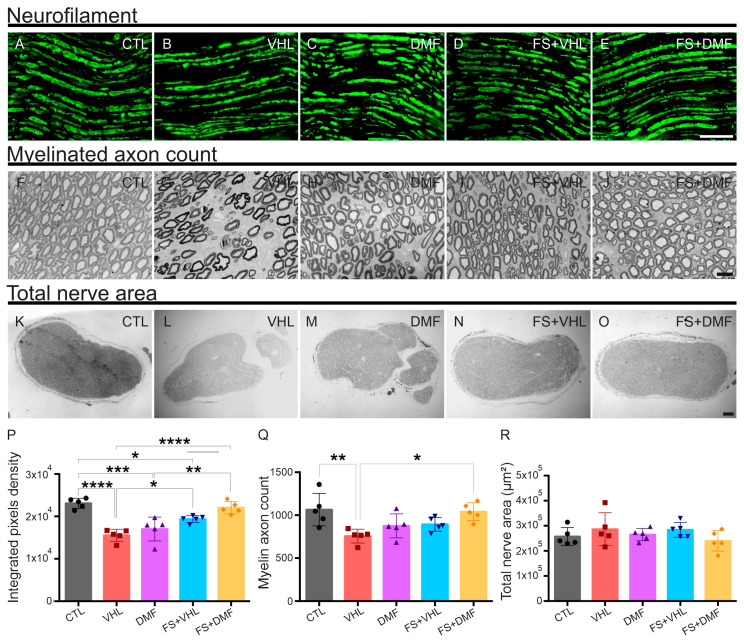
Nerve preservation analysis 12 weeks after injury. (**A**–**E**) Immunostaining analysis of the mid-thigh portion of the sciatic nerve stained with anti-neurofilament; groups that received some therapeutic approach showed a significant increase in labeling compared to the VHL group. 20× magnification; scale bar = 100 μm. (**F**–**J**) Myelin axon counts in cross-sections of the sciatic nerve stained with Sudan black; groups that received some therapeutic approach showed a significant increase in myelinated axon counts compared to the VHL group. 100× magnification; scale bar = 10 μm. (**K**–**O**) Total nerve area in cross-sections of the sciatic nerve stained with Sudan black; no difference was observed between groups. 10× magnification; scale bar = 50 μm. (**P**) Neurofilament quantification (ipsi/contralateral ratio). (**Q**) Myelinated axon count quantification (ipsi/contralateral ratio). (**R**) Total nerve area quantification (ipsi/contralateral ratio). Mean values per group ± standard error of the mean. N = 5. * *p* < 0.05, ** *p* < 0.01, *** *p* < 0.001 and **** *p* < 0.0001.

After 12 weeks of injury, the non-reimplanted groups showed a significant reduction in the immunostaining of the fibers by the neurofilament, which labels intermediate filaments present in the axon cytoskeleton, when compared to the reimplanted and contralateral groups. In parallel with this analysis, we observed that the number of myelinated axons was reduced in both non-reimplanted groups and the FB+VHL group. In contrast, in the FB+DMF group, which received both therapies, there was a recovery in the number of fibers, which approached the values obtained in the CTL group, demonstrating axon sprouting and regrowth towards the target muscles. However, we did not observe a reduction in the area of the sciatic nerves in any of the experimental groups, indicating that the loss in axons in the endoneurial environment was compensated with extracellular matrix synthesis, mostly collagen fibers (Figure 5, Table 6).

### 3.6. Sciatic Nerve Histomorphometry

#### 3.6.1. Myelinated Axon Diameter (FD)

**Figure 6:**
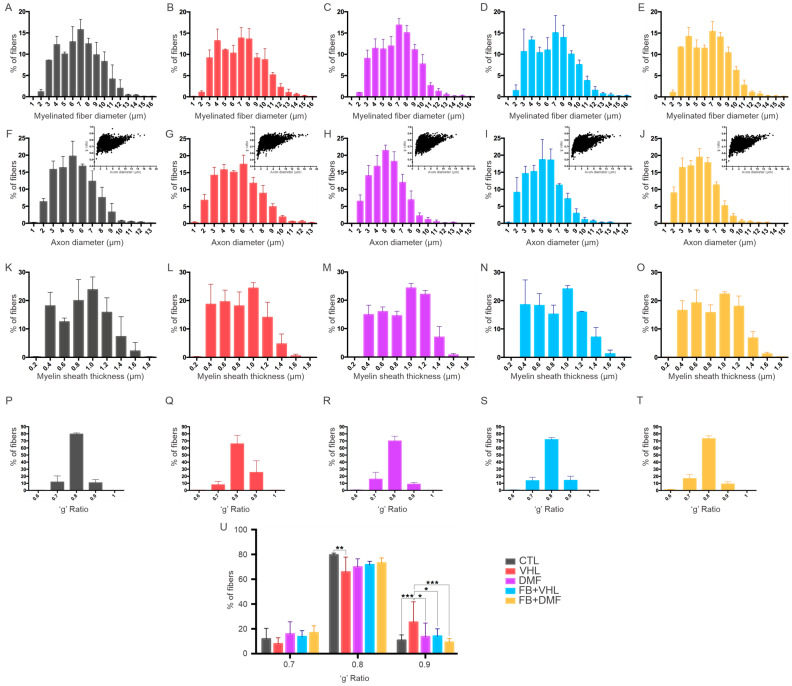
Frequency distribution histogram 12 weeks after injury, referring to (**A**–**E**) myelinated fiber diameter distribution, (**F**–**J**) axon diameter, (**K**–**O**) myelin sheath thickness, (**P**–**T**) ‘g’ ratio, and (**U**) highlighted differences in the percentage of fibers with a ‘g’ ratio of 0.7 to 0.9 in each group. N = 5. * *p* < 0.05, ** *p* < 0.01 and *** *p* < 0.001.

The nerves in the CTL group presented myelin fibers with diameters ranging from 2 μm to 14 μm, distributed in a bimodal manner, with peaks at 4 and 7 μm. The VHL group has a distribution of fibers with smaller dimensions, from 1 to 14 μm, with a predominance of fibers with 4, 7, and 8 μm. The DMF group showed a distribution from 2 to 15 μm, with a predominance of fibers with 7 μm and a reduction in fibers within the 4 μm range. In both SF groups, a distribution of fibers between 2 and 15 μm was observed, distributed in a bimodal way as in the CTL group, with a predominance of fibers within 4 and 7 μm. Thus, the combination of therapeutic approaches was able to preserve the distribution of large and small fibers closer to normal, demonstrating a regenerative effect (Figure 6A–E, Table 7).

#### 3.6.2. Axon Diameter (AD)

We observed that the frequency distribution of axonal diameter among all avulsed groups was similar to each other and differed from that in the CTL group. This similarity is due to the relative increase in the frequency of smaller-diameter fibers, confirming the loss in larger-caliber fibers, compatible with motor axons. The nerves in the CTL group had myelinated axons with minimum diameters ranging from 2 to 12 μm. For the VHL group, myelin axons varied between 1 and 12 μm and between 2 and 11 μm for the groups where there was some therapeutic intervention (Figure 6F–J, Table 7).

#### 3.6.3. Myelin Sheath Thickness (MST)

We observed that the MST frequency distribution among all avulsed groups was similar to each other and differed from that of the control group. The shift in the histogram pattern to the left is highly suggestive, indicating loss in large-caliber fibers, compatible with axons of avulsed motoneurons. Thus, the avulsion-alone group showed an increase in fibers of 0.4, 0.6, and 0.8 μm to almost 20% for each, while, in the other groups, this percentage was lower. Furthermore, all groups that received any of the therapeutic strategies had a higher percentage of fibers within the 1.4 μm (approximately 7%) range compared to the lesion-only group (less than 5%), indicating the presence of larger and more preserved fibers (Figure 6K–O, Table 7).

#### 3.6.4. ’g’ Ratio

The ‘g’ ratio determines whether the myelin sheath thickness is adequate for the axonal diameter so that normal myelinated axons are expected to display 0.7 to 0.8 ratio (in the graph, such an interval is expressed in the column of 0.8). Therefore, the distribution at 0.9 indicates thinly myelinated fibers, possibly with reduced speed in action potential transmission. Interestingly, an increased frequency of fibers in the 0.9 range in the VHL group, but not in the groups that received any of the therapeutic approaches, indicates that regeneration occurred in those groups (Figure 6P–U, Table 7).

### 3.7. Motor Functional Recovery

**Figure 7:**
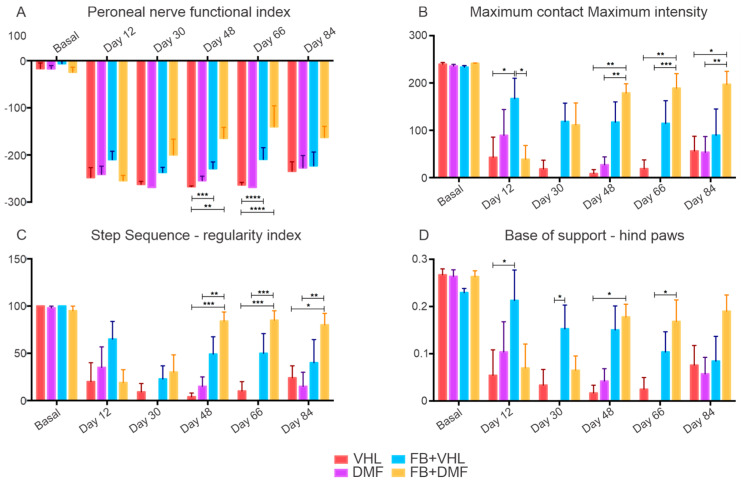
Motor function recovery 12 weeks after injury. Significant motor function improvement was observed from day 48 on all parameters evaluated when lesioned root was reimplanted and animals were orally treated with DMF. (**A**) Functional index of the peroneal nerve. (**B**) Maximum contact maximum intensity, demonstrating the strength with which the particles touch the walkway. (**C**) Step sequence-regularity index, showing improvement in the coordination of the paws in the FB groups. (**D**) The mean base of support of the hind paws, where both FB groups showed restoration of the distance between the paws. N = 5. * *p* < 0.05, ** *p* < 0.01, *** *p* < 0.001 and **** *p* < 0.0001.

Recovery of motor function is a result of neuronal survival and regeneration of axons to the target muscle. As demonstrated before by our group Kempe et al. 2020 [29], we observed that considerable early improvements around day 48 were obtained in the FB+DMF group, while other groups showed poor or delayed recovery regarding nerve functionality, depicted by the peroneal function index. The same positive results were observed regarding the strength (max contact max intensity) and motor coordination (step sequence and base of support hind paws) in which animals almost reached basal values (Figure 7). 

## 4. Discussion

Motor root avulsion is characterized by root pullout from the spinal cord surface, causing disconnection of spinal motoneurons from muscle fibers, resulting in neuronal loss, degeneration of nerve fibers, and paralysis of the target skeletal muscle [14,39,40,41,42]. This experimental model is of relevance since it allows the study of structural changes that occur in the spinal cord microenvironment, such as neuronal degeneration and neurotransmission circuitry modifications, as well as changes in the affected peripheral nerve. After injury, extensive synaptic plasticity occurs in the cell body and proximal dendrites of the motoneuron in response to ventral root avulsion, with retraction, reduction, or elimination of presynaptic terminals apposed to injured neurons [3,9,11,13,43,44,45,46,47,48]. These events may reduce synaptic transmission to low levels or even block it in the acute post-injury period [26,27,49], probably also affecting nearby spinal segments that have not been directly affected by the primary lesion [42,50]. In turn, such an imbalance in synaptic connections impairs voluntary movements and can cause neuropathic pain and/or hyperalgesia. If injury repair and reinnervation do not occur, synaptic transmission can be abolished totally [26,49] and irreversibly [43].

Our results are in line with the literature, both regarding the lesion-only group without any intervention, where synapse loss was noted, as well as in the group where there was reimplantation using FB, restoring the CNS/PNS anatomical continuity [14,41]. Treatment with DMF without reimplantation seems to have a minor effect on the stabilization of spinal circuits, although significant preservation concerning excitatory circuits was previously observed by immunohistochemistry [29]. Therefore, it is clear that the early repair of the lesioned roots is crucial for both motoneuron survival and the preservation of intrinsic spinal circuitry. Further, the early repair positive outcome can be enhanced with the combination of a neuroprotective drug such as DMF. Herein, we demonstrate marked preservation of the spinal circuits by synaptophysin immunolabeling, which was confirmed by the ultrastructural evaluation. This result is strongly related to the reduction in inflammation, possibly due to the reduction in microglial and astroglial activation [29], since these cells have a role in the retraction and elimination of synaptic terminals after injury [51,52,53,54]. This may, in turn, aid in the recovery of motor coordination and function once regenerating motor axons reach the target muscle.

Regarding the ultrastructural organization of synaptic terminals that support the neuronal membrane, we observed that reimplantation alone or associated with DMF seems to provide greater preservation of these terminals and decrease the space between the remaining terminals, possibly preserving the clustering formation. This is in line with a previous work of our group, indicating that the combination of DMF and root reimplantation preserves immunoreactivity to pre-synaptic markers of excitatory and inhibitory inputs present in the motor nucleus [29].

The present work further investigated the synaptic preservation by using transmission electron microscopy, thus providing the typing of the synaptic terminals that remained in apposition to the axotomized alpha motoneuron membrane. Thus, we observed that there is intense elimination of excitatory terminals after injury. This effect is believed to decrease neuronal transmission activity and prevent excitotoxicity caused by glutamate, which can activate apoptotic and necrotic pathways [23,55]. However, all therapies used, alone or in combination, showed preservation of such inputs, demonstrating that they possibly contribute to a pro-regenerative state of the neurons. In fact, Parodi and colleagues [56] pointed out that, in mice with experimental autoimmune encephalomyelitis (EAE), a model for multiple sclerosis, the neuroprotective effect of DMF occurs directly on presynaptic terminals through immunomodulation of glutamate release and indirectly, post-synaptic, through the modulation of microglial function. Kempe and colleagues [29] observed an intense loss in inhibitory inputs through immunostaining with anti-GAD65 after avulsion, and significant preservation of such inputs after reimplantation and DMF treatment. In line with that, the present data based on the typing of the synaptic buttons indicated that they remained close to the values obtained for the uninjured neurons. Thus, the reimplantation by itself or in combination with DMF seems to allow the axotomized neurons to remain wired to the motor nucleus in a similar fashion to the uninjured counterpart.

After a peripheral nerve injury, several events are triggered in order to allow repair and reconnection to the target. Thus, the distal stump develops Wallerian degeneration, with degeneration of the myelin sheath and injured axons. In the case of ventral root avulsion, axons initially regenerate within the CNS and cross the glial scar formed at the edge of the spinal cord, which plays a fundamental role in the failure of central axons to regenerate efficiently as it acts as a physical barrier to their passage. In a second moment, the axons must regenerate towards the reinnervation of the target, through the PNS. Macrophages and Schwann cells assist in these steps, clearing debris and providing interleukins, neurotrophic factors [57,58,59,60], and an extracellular matrix [61], which are fundamental to the regenerative process. Thus, lesioned root repair with FB and pharmacological treatment with DMF has the potential for inducing the regeneration of nerve fibers due to their neuroprotective and immunomodulatory properties. In particular, through the control of gliosis [29], reduction or decrease in the glial scar formation can be achieved, combined with stimulation of Schwann cells in the nerve, allowing enhanced regeneration of myelinated fibers.

The present results show a marked decrease in the number of large, myelinated axons after ventral root avulsion. When therapeutic interventions were used, there was a restoration of a number of fibers, indicating the occurrence of regeneration of motor axons. Thus, due to the ability of DMF to act on Schwann cells [56,62,63], we suggest that the treatment leads to protection and substantial regenerative response. The FB, in turn, by re-establishing the CNS/PNS connection, allows a similar positive outcome [14,64]. Therefore, when both therapies were combined, more robust results in protection and regeneration were observed. 

Of note, it has already been shown that the administration of neurotrophic factors after spinal cord injury in rodents and primates [65,66] promotes axonal regeneration, inducing increased expression of neurofilaments necessary for the elongation of the regrowing axons [67]. Thus, our results demonstrated greater expression of neurofilament after the association of therapies when compared to that observed in animals that did not receive treatment. This is probably related to the fact that DMF treatment induces the production of neurotrophic factors, such as BDNF, GDNF, and NT3 [68]. The present morphometric results show that the frequency distribution pattern of fibers changes between uninjured and injured nerves, demonstrated by the large increase in thin fibers with a ‘g’ ratio of 0.9, which is in part the result of the interactions between Schwann cells and growing axons [69,70,71]. All groups that received any of the therapies presented improved “g” ratio frequency distribution, close to the value found in nerves that were not injured. Together with the “g” ratio, the myelin sheath thickness (MST) is a parameter closely related with Schwann cell activity. Our results indicate that the association between early repair with FB and DMF accelerates the regrowth and myelination of large-diameter fibers, with their distribution being closer to the uninjured nerve. Regarding the diameter of the axons, fibers with smaller dimensions after injury were observed in all experimental groups when compared to the uninjured side, which is compatible with what is described in the literature [72]. Regarding large fibers, since the ventral root avulsion does not affect sensitive axons, the presence of large axons is expected even in the avulsion-alone group. Overall, the frequency distribution of such fibers showed improvements after reimplantation and DMF therapy.

Although DMF alone induces nerve fiber regeneration, the long-lasting effects observed herein were very robust. DMF increases the expression of the enzyme HO-1 (heme oxygenase), which has cytoprotective and anti-inflammatory effects, participating in the anti-oxidative pathway [63,73,74,75], which may support the long-term regenerative response. Szepanowski and colleagues [76], using a model of sciatic nerve crush, related the increase in the NRF2 factor with the increase in the HO-1 enzyme and glutathione. These results are in line with what was observed in the current work, where greater recovery of fibers, combined with a close-to-normal ‘g’ ratio, was achieved.

The functional improvement suggests that the repaired nerve fibers were successful in crossing the glial scar, which functions as a physical barrier at the interface between the CNS and the PNS. Furthermore, peripheral regeneration depends on the long-term survival of the injured neuron and the entry of regenerating axons into the endoneurial tubes, which provide guided axon growth [77]. Thus, given that both DMF and FB have neuroprotective and immunomodulatory potential, as previously observed by Kempe and colleagues [29], the enhanced motor recovery observed herein demonstrates that newly formed motor axons reach target muscles and restore, to a certain degree, the motor coordination.

DMF is capable of providing neurotrophic factors, such as BDNF, GDNF, and NT3 [68,78], these neurotrophic molecules being directly related to axonal elongation after peripheral nerve injury and during development [79] and also having been associated with motor recovery after cerebral ischemia [80]. Also, Szepanowski and colleagues [76] related the improvement in the grip strength test with the re-establishment of the ‘g’ ratio, which indicates nerve fiber functionality, as observed in our work. Furthermore, these authors observed an increase in the expression of antioxidant enzymes and the NRF2 factor, showing a decrease in malondialdehyde, a marker for lipid stress [68,76]. In addition, the control or reduction in inflammatory responses provides a pro-regenerative environment that allows improvements in functional performance [29,81,82].

## 5. Conclusions

Pharmacological treatment with DMF associated with lesioned root reimplantation showed neuroprotective and anti-inflammatory capacity. Thus, we obtained a preservation of spinal circuits combined with a close-to-normal clustering of inputs in direct contact with alpha motoneurons, which may have contributed to the motor recovery over time. Therefore, the combined therapy of DMF with root replantation with fibrin biopolymer may be further studied, aiming at future therapeutic application.

## Figures and Tables

**Table 1:** Antibodies used for the immunofluorescence assay. Details regarding the supplier, host, product catalog number, and concentration used are provided.

Antibody	Supplier	Host	Product Cat No.	Concentration
Synaptophysin	Synaptic Systems (Göttingen, Germany)	rabbit	NBP2-25170	1:1000
Neurofilament	Millipore (Burlington, MA, USA)	rabbit	AB1989	1:2000
NeuN	Millipore	mouse	MAB377	1:1500
Alexa 488	Jackson (West Grove, PA, USA)	rabbit	711-545-152	1:400
Alexa 594	Jackson	mouse	715-585-150	1:400

**Table 2:** Overall synapse preservation in the neuropile (20× magnification) and in close apposition to spinal motoneurons (40× magnification). The integrated density of pixels, ratio ipsi/contralateral.

Groups	Microenvironment	Motoneuron Coverage
VHL	0.432 ± 0.031	0.412 ± 0.043
DMF	0.635 ± 0.037	0.541 ± 0.061
FB+VHL	0.750 ± 0.044	0.738 ± 0.043
FB+DMF	0.801 ± 0.040	0.741 ± 0.027

**Table 3:** Ultrastructural quantification of alpha-motoneuron regarding neuronal perimeter, total synapse coverage, and number of buttons/100 μm of neuronal membrane.

Groups	Neuronal Perimeter (μm)	Total Synapse Coverage (%)	Number of Buttons/100 μm of Neuronal Membrane
CTL	140.0 ± 7.2	63.03 ± 1.82	40.36 ± 2.05
VHL	105.0 ± 9.7	43.06 ± 4.38	23.22 ± 2.37
DMF	135.1 ± 5.2	51.7 ± 3.72	30.80 ± 2.75
FB+VHL	148.9 ± 3.1	64.87 ± 1.51	36.20 ± 1.26
FB+DMF	141.5 ± 4.2	63.32 ± 2.24	35.64 ± 1.32

**Table 4:** Ultrastructural quantification of alpha-motoneuron regarding type F, S, and C total synapse coverage and type F, S, and C number of buttons/100 μm of neuronal membrane.

Groups	Total Synaptic Coverage (%)			Number of Boutons/100 μm		
	F	S	C	F	S	C
CTL	51.98 ± 1.95	7.10 ± 0.89	3.94 ± 0.66	35.55 ± 2.21	3.72 ± 0.46	1.45 ± 0.15
VHL	36.08 ± 4.20	1.63 ± 0.86	5.34 ± 1.21	23.80 ± 3.14	0.80 ± 0.24	1.50 ± 0.26
DMF	40.13 ± 2.82	6.33 ± 1.61	5.22 ± 1.30	26.30 ± 2.08	2.55 ± 0.66	1.40 ± 0.22
FB+VHL	54.14 ± 1.66	4.54 ± 0.79	6.17 ± 0.73	32.40 ± 1.27	2.20 ± 0.43	1.60 ± 0.16
FB+DMF	43.35 ± 3.42	5.12 ± 0.87	5.14 ± 0.68	26.45 ± 2.06	2.60 ± 0.38	1.35 ± 0.13

**Table 5:** Quantitative values of incomplete or insufficient regeneration of alpha motoneurons, regarding total synaptic coverage, number of buttons/100 μm of neuronal membrane, and the total number of intervals.

Groups	Total Synaptic Coverage (%)	Number of Buttons/100 μm of Neuronal Membrane	Number of Intervals
CTL	63.03 ± 1.82	40.36 ± 2.05	44.73 ± 2.05
VHL	43.06 ± 4.38	23.22 ± 2.37	24.30 ± 3.40
FB+DMF	30.96 ± 3.31	17.83 ± 2.38	20.17 ± 1.90

**Table 6:** Quantitative values regarding neurofilament immunostaining, myelinated axon counts, and total nerve area.

Groups	Neurofilament Immunostaining	Myelinated Axon Counting	Total Nerve Area (μm^2^)
CTL	23,105 ± 533	1064 ± 84	257,097 ± 15,843
VHL	15,526 ± 642	756 ± 36	286,198 ± 29,407
DMF	17,021 ± 1254	876 ± 62	264,682 ± 10,953
FB+VHL	19,361 ± 362	893 ± 35	284,019 ± 13,227
FB+DMF	22,052 ± 642	1041 ± 46	239,858 ± 18,166

**Table 7:** Sciatic nerve histomorphometry. Mean values of fiber diameter (FD), axon diameter (AD), myelin sheath thickness (MST), and ‘g’ ratio in the different experimental groups, 12 weeks after injury. Values are presented as the mean ± standard error of the mean.

Groups	FD (μm)	AD (μm)	MST (μm)	‘g’ Ratio
CTL	6.193 ± 0.033	4.658 ± 0.026	0.767 ± 0.004	0.750 ± 0.0005
VHL	6.418 ± 0.043	4.974 ± 0.036	0.721 ± 0.004	0.768 ± 0.0008
DMF 15	6.237 ± 0.035	4.670 ± 0.028	0.783 ± 0.004	0.746 ± 0.0007
FB+VHL	6.199 ± 0.038	4.673 ± 0.030	0.762 ± 0.004	0.749 ± 0.0007
FB+DMF	5.997 ± 0.033	4.462 ± 0.026	0.767 ± 0.004	0.739 ± 0.0006

## Data Availability

The datasets generated during the current study are available from the corresponding author upon reasonable request.
